# Rehabilitation and Return to Play Protocols Following Osteochondral Autograft and Allograft Transplantation for Knee Chondral Lesions in Athletic Populations

**DOI:** 10.1007/s12178-026-10033-y

**Published:** 2026-05-22

**Authors:** S. Ryan Pierson, Richard M. Silverman, Brock Knapp, Robert H. Brophy

**Affiliations:** Department of Orthopaedic Surgery, Washington University School of Medicine, St. Louis, MO USA

**Keywords:** Osteochondral allograft, Osteochondral autograft, Rehabilitation, Return to play, Knee chondral lesion, Articular cartilage

## Abstract

**Purpose of Review:**

Osteochondral autograft transplantation (OATS) and osteochondral allograft transplantation (OCA) are established cartilage restoration procedures for symptomatic chondral and osteochondral defects of the knee in athletes. Postoperative rehabilitation is central to graft healing and incorporation, functional recovery, and safe return to play (RTP). This review synthesizes contemporary evidence on rehabilitation after OATS and OCA, including weight-bearing progression, bracing, range of motion, blood flow restriction training, and RTP criteria.

**Recent Findings:**

Systematic reviews and survey studies report substantial variability in rehabilitation protocols, including weight-bearing timelines, bracing duration, continuous passive motion utilization, and RTP criteria. Few published protocols incorporate objective functional testing to guide RTP. Criteria-based frameworks that individualize progression by graft type, lesion location, and functional milestones are increasingly advocated, although supporting evidence remains limited. Blood flow restriction training may help preserve strength early after surgery, but data specific to OATS and OCA remain sparse. Return to play is commonly reported after both procedures, with earlier timelines more frequently reported after OATS than OCA.

**Summary:**

Rehabilitation after OATS and OCA requires balancing early graft protection with progressive restoration of motion, strength, and sport-specific capacity. Current evidence demonstrates wide protocol heterogeneity and continued reliance on time-based milestones, with underuse of objective RTP criteria. Standardized, criteria-driven pathways and multidisciplinary decision-making may improve consistency and optimize outcomes in athletic populations.

## Introduction

Chondral and osteochondral knee lesions are common among athletes, with full-thickness focal defects reported in approximately 36% of athletic populations undergoing arthroscopic evaluation [1, 2]. These injuries pose a clinical challenge, as articular cartilage is avascular with limited intrinsic healing capacity [1, 3]. Untreated focal chondral defects may progress to osteoarthritis, resulting in persistent pain and functional limitations compromising athletic participation [1].

For appropriately selected patients, cartilage restoration procedures aim to reconstitute the articular surface and preserve joint function [1, 4]. Osteochondral autograft transplantation (OATS) transfers cylindrical osteochondral plugs with mature hyaline articular cartilage from a limited femoral weight-bearing region into the defect, typically reserved for focal lesions < 2–3 cm^2^ [5, 6]. Osteochondral allograft transplantation (OCA) uses fresh, size-matched donor osteochondral tissue to maintain chondrocyte viability and is commonly applied to larger defects (> 2–3 cm^2^), following failed prior cartilage procedures, or lesions with substantial subchondral bone involvement [5, 7, 8]. Both procedures demonstrate favorable outcomes and successful return to play (RTP) is commonly reported in athletes, although rates vary across studies [6, 9, 10].

Postoperative rehabilitation is central to clinical success, influencing graft incorporation, articular surface integrity, and RTP [11, 12]. Despite the importance of rehabilitation, systematic reviews and institutional surveys demonstrate heterogeneity in protocols, with variability in weight-bearing progression, bracing strategies, range of motion (ROM) guidelines, and RTP criteria [11–13]. Most protocols remain time-based and rarely incorporate objective functional testing to guide RTP decisions [11, 12].

The purpose of this review is to synthesize the current literature on rehabilitation protocols and RTP following OATS and OCA for knee chondral lesions in athletic populations. Emphasis is placed on weight-bearing progression, ROM guidelines, bracing strategies, blood flow restriction (BFR) training, RTP timelines, and RTP criteria. In protocols where the evidence is sparse or conflicting, the senior author’s institutional approach is described.

## Surgical Overview and Biological Considerations

### Osteochondral Autograft Transplantation (OATS)

OATS entails harvesting cylindrical osteochondral plugs from low–contact-pressure donor site—most commonly the superolateral ridge of the lateral femoral condyle or intercondylar notch—with subsequent press-fit implantation into a prepared recipient socket at the defect site [5]. Because the graft consists of autologous hyaline cartilage with subchondral bone, immediate structural continuity is maintained [5, 6].

Graft biomechanics evolve during the early postoperative period. In a rabbit model evaluating osteochondral graft-recipient constructs after OATS, Nakaji et al. demonstrated cartilage stiffness was comparable to native cartilage immediately after implantation but declined significantly at postoperative weeks 1, 3, and 8 before normalizing by week 12, coinciding with subchondral bone remodeling [14]. Similarly, when analyzing short-term changes after OATS in a goat model by Lane et al., bone plugs retained 95% chondrocyte viability at 12 weeks; however, the subchondral interface demonstrated increased trabecular density and stiffness, reflecting an active remodeling phase [15]. At longer-term follow-up, Pareek et al. reported successful outcomes in 72% of patients undergoing OATS at a mean follow-up of 10.2 years, supporting technique durability [6].

## Osteochondral Allograft Transplantation (OCA)

OCA utilizes fresh, size-matched donor osteochondral tissue, providing mature hyaline cartilage supported by a bone scaffold [5, 7]. A principal advantage of OCA is resurfacing large defects (> 2–3 cm²) without donor-site morbidity [5, 7, 8]. However, the allograft bone undergoes creeping substitution, in which the donor osseous scaffold is gradually resorbed and replaced by host bone [7, 8]. Donor chondrocyte viability is critical to graft success, and preservation of viable chondrocytes within transplanted tissue determines cartilage durability [16–18]. In a canine model evaluating donor chondrocyte viability during implantation with respect to functional success of femoral condylar OCAs, Cook et al. found all successful OCAs had > 70% chondrocyte viability at the time of implantation, while no graft with chondrocyte viability < 70% during implantation was associated with successful outcomes [16]. As osseous integration is prolonged compared with OATS, early rehabilitation after OCA is generally more conservative, including delayed progression to impact activities [7, 8, 19].

## Influence of Lesion Location and Concomitant Procedures

As the biomechanical environment differs between femoral condyle and patellofemoral lesions, rehabilitation must account for lesion location [3, 20]. Lesions in the femoral weight-bearing region are primarily exposed to compressive forces, whereas patellar and trochlear lesions experience shear forces during knee flexion as the patella engages the trochlear groove [3]. These differences influence weight-bearing, bracing, and ROM protocols [3, 11]. Concomitant procedures—including high tibial osteotomy, meniscal allograft transplantation, or ligament reconstruction—may further modify rehabilitation timelines and require individualized consideration [21, 22].

## General Structure of Rehabilitation Protocols

### Phased Rehabilitation Models

Rehabilitation following OATS and OCA is structured into phases progressing from graft protection to functional recovery and RTP [12, 20, 23]. Although the precise number of phases and duration vary across protocols, common objectives include control of pain and effusion, gradual restoration of ROM, advancement of weight bearing, strength and neuromuscular recovery, and sport-specific activity reintroduction [12, 20, 23]. A five-phase protocol was described by Haber et al. and Patel et al. [20, 24] following OCA. Haber et al. outlined a time-based approach in which Phase I (weeks 0–8) emphasizes graft protection with non–weight bearing (NWB) with unrestricted ROM; Phase II (weeks 8–12) introduces progressive weight bearing, gait normalization, and cardiovascular exercise; Phase III (weeks 12–16) develops functional strengthening; Phase IV (weeks 16–20) further advances lower extremity strength and full weight bearing cardiovascular exercises; and Phase V (beyond 20 weeks) initiates return-to-sport activities [20]. In contrast, Patel et al. described a multidisciplinary protocol, with Phase I (weeks 0–2) focusing on pain and effusion control using protected partial weight bearing, postoperative bracing for graft protection, and early quadriceps activation including BFR consideration; Phase II (weeks 2–6) implementing stepwise progression of weight bearing and gait normalization; Phase III (weeks 6–12) advancing strengthening and initiating cardiovascular conditioning; Phase IV (weeks 12–20) targeting eccentric strength development, movement assessment, and modified sport-specific activity reintegration; and Phase V (beyond 20 weeks) incorporating plyometrics, agility training, and a criteria-based return to competition [24].

### Variability Across Institutions

Despite these frameworks, standardization remains limited. Crowley et al. analyzed 35 rehabilitation protocols from 155 US academic orthopaedic programs and found only 16 programs (10%) published OATS or OCA protocols [11]. Among these protocols, variation was observed in weight-bearing progression, bracing, strengthening, proprioceptive training, and RTP criteria [11]. Garcia-Mansilla et al. similarly documented heterogeneity in physical therapy protocols for OCA of the femoral condyles, reinforcing the need for evidence-based standardization [13]. Kane et al. surveyed 76 surgeons through the Metrics of Osteochondral Allografts (MOCA) study group and reported weight-bearing restrictions ranged from immediate NWB progressing to full weight bearing by 12 weeks to immediate weight bearing as tolerated (WBAT), with higher-volume surgeons (> 20 OCA procedures per year) tending to adopt less restrictive protocols [25]. A recent MOCA database study by Prigmore et al. compared immediate unrestricted WBAT with restricted toe-touch weight-bearing protocols after femoral OCA and found immediate WBAT was noninferior with respect to patient-reported outcome measures (PROMs), suggesting less restrictive rehabilitation may be safe in select patients [26].

## Weight-Bearing Progression

### OATS

Following OATS, strict NWB is commonly recommended for two weeks to allow for subchondral bone remodeling [3, 14, 15, 27]. Partial weight bearing is typically introduced between weeks two and six, with progression to full weight bearing by approximately eight weeks, consistent with the expected subchondral bone integration timeline [3, 11]. Crowley et al. reported across published academic protocols that the mean time to permitted WBAT following OATS was 5.2 weeks (range, 0–8 weeks), with a mean full weight bearing goal of 6.9 weeks (range, 0–9 weeks) [11].

### OCA

Weight-bearing restrictions are generally more conservative after OCA due to the prolonged period of osseous incorporation [7, 20]. Haber et al. recommended NWB for eight weeks, with advancement to full weight bearing between weeks 8 and 12 [20]. Stark et al. found across 62 studies encompassing 3451 knees, the most common timeframe for WBAT following OCA to be six weeks [12]. Crowley et al. reported a mean WBAT at 6.2 weeks (range, 0–8 weeks) and a mean goal time to full weight bearing of 7.7 weeks (range, 6–12 weeks) after OCA [11]. Patel et al. described a more graduated progression in professional athletes, beginning with 20% flat-foot weight bearing during weeks 0 to 2, advancing to 50% at weeks 2 to 4 with crutches, and reaching WBAT by weeks 4 to 6 [24]. Prigmore et al. reported immediate unrestricted WBAT following femoral OCA did not result in inferior PROMs or failure rates at a mean follow-up exceeding two years [26]. Kane et al. further noted surgeons with greater experience performing femoral OCAs tended to adopt less restrictive weight-bearing protocols, suggesting clinical experience may favor earlier mobilization with the recognition that rehabilitation for graft sites such as patellofemoral or tibial plateau needs to be tailored to surgeon and patient-specific needs [25].

### Lesion-Specific Considerations

Weight-bearing protocols should consider lesion location [3]. At the senior author’s institution, the following location-specific approach for OATS and OCA is employed:

- **Femoral condyle lesions**: NWB for six weeks followed by progression to WBAT over one to two weeks. This approach aligns with the majority of published protocols and reflects the need to protect condylar grafts from compressive forces inherent to weight-bearing during early osseous integration [11, 12]. Kane et al. reported 52% of surgeons in the MOCA survey recommended immediate NWB after femoral OCA, transitioning to full weight bearing between 6 and 12 weeks [25].
- **Patellar lesions**: WBAT in a brace locked in extension for six weeks postoperatively. This strategy is based on the biomechanical principle that patellar lesions are not exposed to axial compressive loads during extension weight bearing and therefore do not require strict NWB restrictions [3]. Multiple published protocols support early weight bearing in extension for patellofemoral lesions, as locking the brace prevents patellar engagement with the trochlear groove and limits shear forces across the graft site [3, 11, 12].
- **Patellofemoral lesions (trochlear/combined)**: WBAT with the brace locked in extension for six weeks, followed by gradual unlocking to allow 0° to 90° of flexion in the brace until adequate quadriceps control is achieved. Discontinuation of the brace typically occurs two to four weeks thereafter. This approach balances graft protection from shear forces with progressive quadriceps activation, a prerequisite for safe brace removal [3, 20].

Prolonged NWB carries inherent risks, including muscle atrophy, articular cartilage thinning, decreased proteoglycan synthesis, arthrofibrosis, and venous thromboembolism (VTE) [3, 23, 28]. A study evaluating the incidence of VTE after anterior cruciate ligament reconstruction (ACLR) found NWB status 1 week after surgery was a statistically significant risk factor [28]. These risks must be weighed against the need for graft protection when individualizing rehabilitation progression. Anticoagulation should be considered when protected or NWB is indicated, specifically in patients with comorbidities such as known hypercoagulable factors or oral contraceptives [28].

### Range of Motion Guidelines

#### General Principles

Most published rehabilitation protocols restrict knee flexion immediately after OATS/OCA surgery while encouraging early motion within the first two weeks to prevent arthrofibrosis and maintain cartilage nutrition through synovial fluid circulation [3, 11, 12]. Crowley et al. reported all 24 OATS protocols limited knee flexion in the immediate postoperative period; 92% (*n* = 22 of 24 protocols) permitted 90° flexion at a mean of 1.7 weeks, 75% (*n* = 18 protocols) allowed 110° at a mean of 3.4 weeks, and 88% (*n* = 21 protocols) recommended full flexion at a mean of 7.0 weeks [11]. Similar patterns were reported for OCA protocols, with all protocols restricting flexion initially; 100% (*n* = 17 protocols) permitted 90° at a mean of 1.0 week, 47% (*n* = 8 protocols) allowed 110° at a mean of 4.4 weeks, and all recommended full flexion by a mean of 6.7 weeks [11]. In a systematic review analyzing OCA studies, Stark et al. found 56 of 62 studies (90.3%) specified when ROM was initiated. Among these, 44 studies (71.0%) initiated ROM in the first postoperative week, 9 (14.5%) during the second week, and 3 (4.8%) delayed initiation until the fourth week [12]. Most studies permitted unrestricted ROM once initiated, although 5 (8.9%) described more specific limitations, primarily following patellofemoral OCA [12].

### Continuous Passive Motion (CPM)

Continuous passive motion (CPM) use remains variable [11, 12, 25]. Proposed benefits include enhanced synovial fluid circulation, improved nutrient delivery to the articular cartilage, and reduction of inflammatory mediators associated with immobilization, as supported by basic science models [23]. Crowley et al. reported CPM was recommended in 96% of OATS and 94% of OCA protocols [11], whereas Stark et al. found only 47% of studies in their systematic review reported CPM use after OCA [12]. Kane et al. noted approximately 45% of surveyed surgeons prescribed CPM most of the time or always, while the remainder used it infrequently or not at all [25]. Howard et al. concluded in their systematic review that while basic science evidence supports CPM for maintaining ROM and cartilage health, patient-oriented clinical evidence remains limited [23].

At the senior author’s institution, CPM is initiated from 0°−30° for femoral condyle lesions and advanced 5°−10° daily, with a goal of achieving approximately 120° of knee flexion by six weeks. Although published OATS and OCA rehabilitation protocols emphasize early, progressive restoration of motion, specific daily CPM advancement schedules are scarcely detailed in the literature [3, 11, 12, 23]. For patellofemoral lesions, the senior author restricts CPM to 0°−30° during the early postoperative period to limit patellar engagement within the trochlear groove to reduce shear forces across the graft site [3].

### Bracing Strategies

Bracing practices vary considerably across rehabilitation protocols [11]. Crowley et al. reported 88% of OATS protocols and 100% of OCA protocols recommended immediate postoperative bracing, commonly locked in extension during ambulation [11]. Unlocking of the brace typically occurred at a mean of 3.0 weeks (range 0–7) for OATS and 2.8 weeks (range 1–7) for OCA. The duration of brace use averaged 5.7 weeks for OATS and 6.5 weeks for OCA [11].

### Lesion-Specific Bracing: Institutional Approach

At the senior author’s institution, OATS and OCA bracing strategies are tailored to lesion location:

- **Femoral condyle lesions**: No brace is routinely used. Likewise, Tyler and Lung note bracing is not typically performed for femoral condyle lesions [3]. Graft protection is achieved primarily through weight-bearing restrictions rather than external immobilization. Although Haber et al. described the use of knee bracing for eight weeks following femoral OCA [20], early controlled ROM is simultaneously initiated in that protocol, suggesting the brace serves as an adjunct to rehabilitation rather than the primary means of graft protection. Kane et al. further demonstrated variability in postoperative practices [25].
- **Patellofemoral lesions**: Brace locked in full extension for four to six weeks, until appropriate quadriceps activation is achieved [3, 11, 12]. This prevents patellar engagement with the trochlear groove during knee flexion [3]. Crowley et al. found all protocols specifying lesion location recommended immediate immobilization for patellofemoral lesions [11]. Tyler and Lung similarly advocated for a knee orthosis locked in extension during ambulation [3]. Across protocols, the most common criterion for brace discontinuation is the ability to perform a straight leg raise without extensor lag, reflecting adequate quadriceps control [11, 12].

The divergence in bracing strategies between condylar and patellofemoral lesions is supported by biomechanical principles. Namely, the femoral condyle is subjected primarily to compressive loads, whereas the patellofemoral articulation experiences shear forces that are mitigated by restricting knee flexion [3, 6].

### Strengthening Considerations and Blood Flow Restriction Training

#### Early Strengthening and Quadriceps Preservation

Preservation of quadriceps strength is a key rehabilitation goal following OATS and OCA [11, 12]. Both OATS and OCA protocols underscore early motion and strengthening to reduce postoperative stiffness and muscle weakness, reflecting the adverse effects of prolonged immobilization rather than reliance on time alone for recovery [11, 12]. Accordingly, most published protocols include early initiation of quadriceps/hamstring sets, straight-leg raises, and hip abduction and adduction exercises, although strengthening activity start times vary across protocols [11, 12]. Patel et al. emphasized early quadriceps activation and a progressive kinetic-chain approach in their five-phase rehabilitation protocol for professional athletes, with functional demands advanced as criteria-based milestones are achieved [24]. Crecelius et al. similarly highlighted early quadriceps rehabilitation and strict adherence to prescribed protocols as integral to optimize outcomes following articular cartilage surgery of the knee [29].

### Blood Flow Restriction Training

BFR training has emerged as an adjunctive modality that may attenuate muscle atrophy and promote hypertrophy and strength gains using low external loads, as low as 30% of one-repetition maximum [30]. Hughes et al. demonstrated in a systematic review that BFR training in clinical musculoskeletal rehabilitation settings was more tolerable and resulted in statistically significant improvements in muscle strength compared with low-load exercise alone [30]. Jakobsen et al. evaluated the feasibility of BFR added to exercise in patients with early weight-bearing restrictions following cartilage or meniscus repair and found BFR to be feasible and well tolerated [31]. Evidence on BFR specific to OATS or OCA remains limited [30, 31]. Although theoretical benefits exist during periods when high-load resistance training is contraindicated, randomized controlled trials evaluating osteochondral transplantation patients are lacking [30, 31].

Safety considerations using BFR include concern regarding VTE in the early postoperative period, particularly among NWB patients who have elevated baseline risk related to immobility and recent surgery [32]. Bond et al. reviewed the VTE risk associated with BFR following orthopaedic surgery and concluded the likelihood of BFR directly precipitating a thromboembolic event to be low, given the mechanisms described by Virchow’s triad, with BFR enhancing venous return and fibrinolysis [32]. However, the authors also emphasized VTE risk is markedly elevated during the first six postoperative weeks and the possibility of mobilizing a pre-existing asymptomatic thrombus cannot be excluded [32]. As no universal guidelines exist, clinicians should assess individual VTE risk factors before initiating BFR [32, 33]. This cautious approach aligns with the practice at the senior author’s institution, where BFR is used selectively during the early NWB phase and guided by formal risk stratification; it is contraindicated in patients with prior thromboembolic events, significant cardiovascular disease, poorly controlled hypertension, hematologic disorders, compromised soft tissues such as infection, and introduced cautiously in the setting of persistent swelling, delayed wound healing, or other factors that may elevate VTE risk [32, 33].

### Return to Play Timelines and Rates

#### Comparative Timelines

Return to play (RTP) timelines differ between OATS and OCA, reflecting distinct procedural healing trajectories [9, 10]. In a meta-analysis of 2549 athletes undergoing cartilage restoration surgery, Krych et al. reported OATS was associated with earlier mean reported RTP timelines and a higher rate of return to preinjury athletics than OCA, microfracture, and autologous chondrocyte implantation, although heterogeneity existed across studies [9]. In a systematic review of RTP after OCA, Touhey et al. reported 72% of athletes achieved successful RTP after OCA, with 84% returning at an equal or higher level at a weighted mean of 11.1 months [34].

Several studies have evaluated OCA in high-demand athletic populations. Allahabadi et al. reported RTP in 73% of 15 professional athletes at a mean of 1.22 ± 0.4 years, with an 88% return rate among those undergoing isolated primary OCA; among athletes who returned, 91% resumed competition at the same or higher level [35]. Balazs et al. found 80% of elite collegiate and professional basketball players returned to competition at a median of 14 months following OCA [36]. McCarthy et al. reported an adjusted RTP rate of 77% among competitive high school, intercollegiate, and/or professional athletes at a mean of approximately eight months after OCA, noting social factors such as graduation were more frequently cited than persistent pain as reasons for not returning to play [21]. Cook et al. evaluated outcomes after large single-surface, multisurface, and bipolar shell osteochondral allograft transplantation and reported 68% of athletes returned to sport at a median of 16 months, with a combined revision and failure rate of 10% [37]. Notably, the authors reported that noncompliance with the prescribed rehabilitation protocol was associated with a 15.5-fold increased risk of revision or graft failure compared with patients who adhered to postoperative recommendations [37].

#### Return to Play Criteria

##### Predominance of Time-Based Protocols

A major limitation of the literature is the continued reliance on time-based, rather than criteria-based, RTP frameworks [11, 12]. Stark et al. reported in a systematic review only 21% of OCA studies specified RTP criteria, which were predominantly time based [12]. Crowley et al. similarly found functional testing to be recommended in RTP criterion in only 8% of OATS protocols and 6% of OCA protocols [11]. This contrasts with ACLR rehabilitation, where criteria-based RTP frameworks incorporating strength and functional testing are widely used [38].

#### Objective Criteria

Typical objective RTP criteria following OATS or OCA include:

- Quadriceps strength *≥* 90% limb symmetry index (LSI) on isokinetic testing [24].
- Hop testing with ≥ 90% LSI [24].
- Assessment of movement symmetry and balance [24].
- Controlled single-leg squat performance [24].
- Completion of sport-specific functional tasks [24, 39].

Patel et al. described a criteria-based RTP framework following OCA, in which progression to the RTP phase required ≥ 90% LSI on isokinetic strength testing, acceptable single-leg squat mechanics, and clearance through a structured sport-specific functional assessment [24]. Myer et al. proposed a criteria-based rehabilitation algorithm following ACLR, incorporating dynamic assessment of limb strength, functional stability, bilateral symmetry, postural control, power, endurance, agility, and sport-specific technique [38]. This model has been suggested as a useful template for cartilage restoration rehabilitation as well [38].

#### Subjective Criteria

Subjective measures complement objective testing in RTP decision-making and commonly include:

- Performing functional activities without pain [11, 24].
- Confidence and psychological readiness [24, 39].
- Patient-reported outcomes measures, including the Knee Injury and Osteoarthritis Outcome Score (KOOS), International Knee Documentation Committee (IKDC) score, and Tegner activity scale [21, 24, 35].
- Assessment of movement quality and neuromuscular control by the rehabilitation team [24, 39].

#### Imaging Considerations

Postoperative imaging may assist RTP decision-making, although standardized imaging thresholds for graft incorporation have not been established [25]. Kane et al. reported 66% of surgeons routinely obtained postoperative imaging, most commonly radiographs, followed by magnetic resonance imaging, although imaging findings may have been used for surveillance rather than RTP clearance criteria [9].

### Barriers to Successful Return to Play

Athletes may encounter physical and psychological barriers to RTP following osteochondral transplantation [9, 21, 24, 40]. Physical barriers include persistent pain, inability to meet sport-specific demands, recurrent effusion, and fear of reinjury (kinesiophobia) [9, 21, 24, 40]. Medical and surgical complications, including VTE, symptomatic hardware, graft failure, and the need for reoperation, may further impede RTP [40]. Nonadherence to rehabilitation protocols has been identified as a modifiable risk factor associated with graft failure [41, 42]. Rucinski et al. performed a cohort study analyzing compliance with postoperative protocols after osteochondral and/or meniscal allograft transplantations in the knee and reported that patients who adhered to prescribed rehabilitation protocols were 6.3 times less likely to require allograft revision or conversion to total knee arthroplasty and 7.5 times more likely to achieve a successful outcome at 1 to 3 years postoperatively [41]. In a subsequent study, the same group reported that preoperative counseling with a health behavior psychologist significantly reduced nonadherence rates from 26.7% to 9.8% (*p* = 0.02) [42]. McCarthy et al. also noted social factors, particularly graduation, as being commonly cited as a barrier to RTP among adolescent athletes [21].

### Current Limitations and Future Directions

Several limitations within the existing literature warrant emphasis. Substantial heterogeneity exists in rehabilitation protocols across institutions, limiting comparative effectiveness research and leaving clinicians without clear evidence-based recommendations [11–13]. Reliance on time-based progression remains predominant, despite wide interindividual variability in biological healing and functional recovery [11, 12]. Objective functional testing is infrequently incorporated into RTP decision-making, with fewer than 10% of published protocols recommending such measures [11, 12]. Lastly, few studies have examined the long-term implications of RTP on graft durability, reoperation rates, or progression to arthroplasty. Although Cook et al. reported midterm graft survival exceeding 80% at more than five years following high–chondrocyte-viability OCA, nonadherence to rehabilitation (*p* = 0.0434) and concurrent ligament reconstruction at the time of chondral restoration (*p* = 0.0007) were identified as significant risk factors for failure in adjusted multivariable analyses [43]. The long-term durability of cartilage restoration procedures in athletes exposed to repetitive high-impact loading therefore remains uncertain [43, 44].

Future investigations should prioritize multicenter prospective studies comparing standardized, criteria-based rehabilitation protocols with traditional time-based approaches, incorporating validated functional testing batteries into RTP decision-making, while evaluating the long-term effects of sport resumption on graft survival and joint health [9, 11, 12].

## Conclusions

Rehabilitation following OATS and OCA requires an individualized approach, with consideration given to graft biology, lesion location, concomitant procedures, and patient factors [11, 20, 24]. Both OATS and OCA procedures demonstrate favorable RTP rates, with OATS generally associated with earlier RTP, although variability exists across studies [9, 10, 34]. Despite increasing recognition of the value of criteria-based progression, most protocols remain time-based and rarely incorporate objective functional testing in RTP criteria [11, 12]. Adherence to prescribed protocols represents a primary modifiable determinant of outcome, with noncompliance markedly increasing the risk of graft failure and revision surgery [41, 42]. A multidisciplinary, criteria-driven approach integrating restoration of strength, functional testing with limb symmetry indices, sport-specific readiness assessment, and shared decision-making represents the most evidence-informed framework for optimizing RTP outcomes [24, 38, 39]. Standardization of rehabilitation pathways and development of procedure-specific RTP criteria should therefore remain priorities in future investigations [9, 11, 12].

## Key References

- Prigmore B, Haneberg E, Elias T, Wiedrick J, Ballin J, Cole BJ, et al. Comparison of Patient-Reported Outcomes for Immediate Unrestricted Weightbearing Versus Restricted Rehabilitation Protocols After Osteochondral Allograft Transplantation to the Distal Femur. Orthopaedic Journal of Sports Medicine. 2024;12:23259671241264856. https://doi.org/10.1177/23259671241264856.
  - ○ Retrospective cohort study using the MOCA database demonstrating that immediate unrestricted weight-bearing following distal femoral osteochondral allograft transplantation resulted in patient-reported outcomes and failure rates comparable to those observed with traditional restricted weight-bearing protocols.
- Touhey DC, Beady ND, Tartibi S, Brophy RH, Matava MJ, Smith MV, et al. Return to Sport in Athletes After Osteochondral Allograft Transplantation: A Systematic Review. Am J Sports Med. 2025;53:3275–82. https://doi.org/10.1177/03635465251315492.
  - ○ Systematic review of athletes undergoing osteochondral allograft transplantation reporting a 72% return-to-sport rate at a mean of 11.1 months, with most athletes returning to the same or higher level of competition.
- Crowley SG, Pedersen A, Fortney TA, Swindell HW, Saltzman BM, Popkin CA, et al. Rehabilitation Variability Following Osteochondral Autograft and Allograft Transplantation of the Knee. Cartilage. 2022;13:19476035221093071. https://doi.org/10.1177/19476035221093071.
  - ○ Analysis of rehabilitation protocols from U.S. academic orthopaedic programs demonstrating considerable variability in weight-bearing progression, bracing, strengthening, and return-to-sport criteria following OATS and OCA, underscoring the absence of standardized postoperative rehabilitation guidelines.

## Data Availability

No datasets were generated or analysed during the current study.
